# Advances in Molecular Serotyping and Subtyping of *Escherichia coli*^†^

**DOI:** 10.3389/fmicb.2016.00644

**Published:** 2016-05-03

**Authors:** Pina M. Fratamico, Chitrita DebRoy, Yanhong Liu, David S. Needleman, Gian Marco Baranzoni, Peter Feng

**Affiliations:** ^1^Eastern Regional Research Center, Agricultural Research Service, United States Department of Agriculture, WyndmoorPA, USA; ^2^Department of Veterinary and Biomedical Sciences, Pennsylvania State University, University ParkPA, USA; ^3^Division of Microbiology, U.S. Food and Drug Administration, College ParkMD, USA

**Keywords:** *Escherichia coli*, molecular serotyping, subtyping, detection, identification, whole genome sequencing, O-group, H-type

## Abstract

*Escherichia coli* plays an important role as a member of the gut microbiota; however, pathogenic strains also exist, including various diarrheagenic *E. coli* pathotypes and extraintestinal pathogenic *E. coli* that cause illness outside of the GI-tract. *E. coli* have traditionally been serotyped using antisera against the ca. 186 O-antigens and 53 H-flagellar antigens. Phenotypic methods, including bacteriophage typing and O- and H- serotyping for differentiating and characterizing *E. coli* have been used for many years; however, these methods are generally time consuming and not always accurate. Advances in next generation sequencing technologies have made it possible to develop genetic-based subtyping and molecular serotyping methods for *E. coli*, which are more discriminatory compared to phenotypic typing methods. Furthermore, whole genome sequencing (WGS) of *E. coli* is replacing established subtyping methods such as pulsed-field gel electrophoresis, providing a major advancement in the ability to investigate food-borne disease outbreaks and for trace-back to sources. A variety of sequence analysis tools and bioinformatic pipelines are being developed to analyze the vast amount of data generated by WGS and to obtain specific information such as O- and H-group determination and the presence of virulence genes and other genetic markers.

## Introduction

*Escherichia coli* strains are commensal organisms that are part of the normal intestinal microflora of humans and other mammals. The traditional method for identifying *E. coli* uses antibodies to test for surface antigens: the O- polysaccharide antigens, flagellar H-antigens, and capsular K-antigens (described below). There are currently ∼186 different *E. coli* O-groups and 53 H-types, so serotyping is highly complex. There are also many pathogenic groups of *E. coli* that cause disease in humans and animals, including diarrheagenic *E. coli* and the extra-intestinal pathogenic *E. coli* (ExPEC) that cause illness outside of the GI-tract. Diarrheagenic *E. coli* that cause human illness have been classified based on specific sets of virulence genes they carry and the characteristics of the disease they cause (Kaper et al., 2004). These pathotypes include the enteropathogenic *E. coli* (EPEC), enterotoxigenic *E. coli* (ETEC), enteroinvasive *E. coli* (EIEC), enteroaggregative *E. coli* (EAEC), Shiga toxin-producing *E. coli* (STEC), diffusely adherent *E. coli* (DEAC), and adherent invasive *E. coli* (AIEC) that have been associated with Crohn’s disease. There are also hybrid pathotypes, including the enteroaggregative hemorrhagic *E. coli* (EAHEC) that carry STEC- and EAEC-associated virulence genes. As an example, EAHEC serotype O104:H4, an EAEC that acquired the phage that carried the Shiga toxin gene of STEC, caused a large outbreak in 2011 associated with illness in over 3800 individuals and 54 deaths (Frank et al., 2011). Certain *E. coli* serotypes are often associated with specific pathotypes, such as STEC O157:H7 and O103:H21 (Kaper et al., 2004) that are important STEC, often referred to as enterohemorrhagic *E. coli* (EHEC). Therefore, pathogenic *E*. *coli* constitutes a genetically heterogeneous family of bacteria, and they continue to evolve.

Extra-intestinal pathogenic *E. coli* cause illness outside of the gastrointestinal tract, including urinary tract infections, meningitis, pneumonia, septicemia, and other types of infections (Russo and Johnson, 2003; Smith et al., 2007). ExPEC that cause illness in poultry are known as avian pathogenic *E. coli* (APEC). Avian colibacillosis caused by APEC is a major cause of morbidity and mortality associated with economic losses in the poultry industry throughout the world. The human gut is a reservoir for ExPEC that cause human illness. When ExPEC leave the GI tract and infect other parts of the body such as the urinary tract, the blood, or the lungs, illness results (Smith et al., 2007). Animals, particularly, poultry and poultry products (eggs), pork/pigs, and beef/cattle, and also companion animals may carry ExPEC, and thus, these pathogens may be acquired through the food supply, and zoonotic pathogens may also be acquired via contact with animals (Vincent et al., 2010; Nordstrom et al., 2013; Mitchell et al., 2015; Singer, 2015). Investigations of community-acquired UTI and outbreaks of UTI suggested common point sources, such as contaminated food products (Nordstrom et al., 2013). Indeed, high genetic similarity, including antibiotic resistance and virulence gene patterns, between APEC and ExPEC strains causing disease in poultry and humans, respectively, has been observed (Smith et al., 2007; Manges and Johnson, 2012). The ability to differentiate commensal *E. coli* from ExPEC and other pathotypes is important for risk assessment and epidemiological and ecological studies. However, a rapid and reliable typing/identification system or criteria that allows this type of discrimination and that also provides information on the organism’s evolutionary history, fitness, and pathogenic potential has not yet been established. Determining whether an *E. coli* strain is an ExPEC and whether it is pathogenic is based on its source, O:K:H serotype, phylogenetic background, virulence factor profile, and experimental virulence in an animal model. ExPEC belong to specific phylogenetic groups (A, B1, B2, and D) determined based on multilocus enzyme electrophoresis, ribotyping, or by triplex PCR targeting the genes *chuA* and *yjaA* and a particular DNA fragment known as TSPE4.C2. ExPEC strains belonging to phylogenetic groups B2 and D show higher virulence in humans (Clermont et al., 2000; Smith et al., 2007). It has become evident that certain ExPEC lineages or clonal groups are responsible for a large fraction of human extraintestinal *E. coli* infections, and these lineages are becoming increasingly multi-drug resistant (Smith et al., 2007; Manges and Johnson, 2012).

Rapid and accurate molecular methods are critically needed to detect and trace pathogenic *E. coli* in food and animals and for epidemiological investigations to enhance food safety and animal and human health, as well as to minimize the size and geographical extent of outbreaks. As opposed to traditional serotyping using antisera raised against the different *E. coli* O- and H-types, molecular serotyping generally refers to genetic-based assays targeting O-group-specific genes found within the *E. coli* O-antigen gene clusters and the H-antigen genes that encode for the different flagellar types. Although determining the *E. coli* serotype could be considered a component of subtyping (differentiation beyond the species level), methods used for molecular subtyping such as pulsed-field gel electrophoresis (PFGE), multilocus sequence typing (MLST), and whole genome sequencing (WGS) generate a unique “fingerprint” of the bacterium that can be used in outbreak investigations and to determine the source of illnesses. There are many problems associated with traditional serotyping for determining the *E. coli* O- and H-groups. It is costly, labor-intensive and time consuming, cross reactivity of the antisera with different serogroups occurs, antisera are available only in specialized laboratories, batch-to-batch variations in antibodies can occur, and many *E. coli* strains isolated from various sources are non-typeable (Lacher et al., 2014). Thus, molecular serotyping offers alternative methods for *E. coli* serotyping, and furthermore, they can be coupled with assays for specific virulence gene enabling the determination of O- and H-group, pathotype, and the strain’s pathogenic potential simultaneously.

## *E. coli* O-, K-, and H-Antigens

The outer membrane of *E. coli* is composed of lipopolysaccharides (LPS) that includes lipid A, core oligosaccharides, and a unique polysaccharide, referred to as the O-antigen. Loss of the O-antigens results in attenuated virulence suggesting their importance in host–pathogen interactions (Sarkar et al., 2014). Based on the antigenic diversity among the different O-antigens, they have been targeted as biomarkers for classification of *E. coli* since the 1940s (Kaufmann, 1943, 1944, 1947). Later, Ørskov et al. (1977) presented a comprehensive serotyping system for 164 *E. coli* O-groups and developed a typing scheme based on the presence of three principal surface antigens, O-antigens, flagellar H-antigens, and capsular K-antigens. Since few laboratories had capabilities to type the K antigen, serotyping based on O- and H-antigens became the gold standard for *E. coli* typing. Currently, O-groups numbered O1-O188 have been defined, except for O31, O47, O67, O72, O94, and O122 that have not been designated (Ørskov and Ørskov, 1984; Scheutz et al., 2004), and four groups have been divided into subtypes O18ab/ac, O28ab/ac, O112ab/ac, and O125ab/ac, giving a total of 186 O-groups.

The conventional serotyping method is based on agglutination reactions of the O-antigen with antisera that are generated in rabbits against each of the O-groups (Ørskov and Ørskov, 1984). The method is easy to carry out; however, it is laborious and error-prone, and thus, molecular methods are better alternatives for O-typing (Ballmer et al., 2007; Lacher et al., 2014). The genes that encode for O-antigens are located on the chromosome in a cluster designated as the O-antigen gene cluster (O-AGC). These are flanked by two conserved sequences called JUMPstart, a 39 bp-element at the 5′ end (Hobbs and Reeves, 1994), which is downstream of *galF* (UTP-glucose-1-phosphate uridylyltransferase) and *gnd* (6-phosphogluconate dehydrogenase) at the 3′ end. Analysis of the O-AGCs of all *E. coli* O-groups (Iguchi et al., 2015a; DebRoy et al., 2016) showed that the sizes of the O-AGCs and their gene content vary considerably, which results in the variability of O-antigens. O-antigens are composed of 10–25 repeating units of two to seven sugar residues and are processed by three mechanism of which the most common is Wzy (O antigen polymerase) dependent, followed by an ABC transporter dependent system, and the third mechanism, which involves a synthase dependent pathway (Greenfield and Whitfield, 2012) by which the O-antigens are flipped across the outer membrane. The pathways for biosynthesis of the O-AGCs and assembly of O-antigens have been studied extensively (Samuel and Reeves, 2003). Each of the O-antigens that utilize Wzy-dependent pathway carries two unique genes *wzx* (O-antigen flippase) and *wzy* (O-antigen polymerase). Wzx proteins translocates the O-units across the inner membrane, and Wzy polymerizes the O-antigen (Samuel and Reeves, 2003). For the ABC transporter-dependent pathway, *wzm* (O-antigen ABC transporter permease gene) and *wzt* (ABC transporter ATP-binding gene) are involved in O-AGC synthesis. The O-AGCs are composed of nucleotide sugar biosynthesis genes that are involved in the synthesis of O-antigen nucleotide sugar precursors, the glycosyl transferases that transfer the various sugar precursors to form the oligosaccharide, and the O-antigen processing genes described above.

All of the O-AGC clusters have been sequenced, and sequence analyses revealed that some O-AGCs are 98–100% identical (Iguchi et al., 2015a; DebRoy et al., 2016) while others have point mutations or insertion sequences which causes these to type as different serogroups (Liu et al., 2008, 2015). Therefore, there is a need to resolve these discrepancies, merge or eliminate serogroups and to revise the *E. coli* serotype nomenclature (DebRoy *et al., 2*016). Furthermore, many of the *E. coli* O-AGCs have been found to be identical to those of other Enterobacteriaceae members such as *Shigella* and *Salmonella* (Wang et al., 2007). Out of 34 distinct *Shigella* O-antigens, 13 were unique to *Shigella*; however, the other 21 were also found in *E. coli* (Liu et al., 2008). Similarly, out of 46 O-AGCs of *Salmonella*, 24 of were found to be identical or closely related to *E. coli* O-antigens (Liu et al., 2014).

Serology has defined 53 H-flagellar antigens (Ørskov and Ørskov, 1984; Ewing, 1986) that are numbered from H1 to H56, but H-types 13, 22, and 50 are not in use (Ørskov et al., 1975; Centers for Disease Control and Prevention [CDC], 1999). Molecular H-typing methods are based on the sequences of *fliC* gene that encode for the FliC, the flagellar filament structural protein (Wang et al., 2003). The N- and C-terminals of FliC are highly conserved, so different H-types are due to amino acid differences within the central region, which is the surface-exposed antigenic part of the flagellar filament (Namba et al., 1989). Thus, PCR methods developed to distinguish H-types target the variable region of the *fliC* gene (Machado et al., 2000); however, these regions of some H-types such as H1 and H12 and H25 and H28 are very similar, making them difficult to distinguish. However, a two-step PCR method was developed that can distinguish between *fliC*_H1_ and *fliC*_H12_ (Beutin et al., 2015, 2016). Other methods such as Matrix-Assisted Laser Desorption/Ionization Time-of-Flight (MALDI-TOF)-based peptide mass fingerprinting in conjunction with a custom *E. coli* H-antigen data base (Cheng et al., 2014) has been also utilized to distinguish H-types (Chui et al., 2015).

## Methods Used for Subtyping and Molecular Serotyping of *E. coli*

Subtyping methods that allow for differentiation of *E. coli* beyond the species and subspecies level are critical for determining the source of outbreaks and establishing transmission pathways (Eppinger et al., 2011; Frank et al., 2011). Several phenotype-based and genotype-based methods for subtyping *E. coli* are listed in **Table 1**. Phenotypic culture methods, in conjunction with biochemical-based testing, serotyping, phage typing, multilocus enzyme electrophoresis have been used for many years and could be considered gold standard methods; however, they are time and labor intensive and may not be very discriminatory.

**Table 1:** Phenotype- and genotype-based methods for subtyping and molecular serotyping of *E. coli*.

Phenotype-based methods	O-antigen^a^	H-antigen^a^	Virulence-related and other genes	SNPs and other markers	Reference
Immunological O/H typing (serotyping)	X	X			Ørskov et al., 1977
Bacteriophage typing				X	Ahmed et al., 1987; Krause et al., 1996
Multilocus enzyme electrophoresis (MLEE)				X	Ochman and Selander, 1984; Selander et al., 1986; Campos et al., 1994
MALDI-TOF		X		X	Chui et al., 2015
**Genotype-based methods**					
Restriction length polymorphism (RFLP)	X	X	X		Coimbra et al., 2000; Beutin et al., 2005; Moreno et al., 2006; Abbadi and Strockbine, 2007
Luminex-based suspension assay	X				Lin et al., 2011; Clotilde et al., 2015
Amplified fragment length polymorphisms (AFLP)				X	Hahm et al., 2003; Leung et al., 2004
Optical mapping			X	X	Kotewicz et al., 2007, 2008; Miller, 2013
Ribotyping				X	Martin et al., 1996; Carson et al., 2001
Multilocus variable number tandem repeat analysis (MLVA)				X	Hyytiä-Trees et al., 2006; Byrne et al., 2014
Pulsed-field gel electrophoresis			X	X	Krause et al., 1996; Ribot et al., 2006
Multilocus sequence typing (MLST)				X	Eichhorn et al., 2015; Manges et al., 2015
High throughput real-time PCR	X	X	X		Bugarel et al., 2010a,b, 2011a,b; Delannoy et al., 2013; Tseng et al., 2014
Multiplex PCR	X	X	X		Fratamico and DebRoy, 2010; Botkin et al., 2012; Doumith et al., 2012; Fratamico et al., 2014; Iguchi et al., 2015b
Whole genome sequencing and SNP analysis	X	X	X	X	Zhang et al., 2006; Eppinger et al., 2011; Norman et al., 2012, 2015; Joensen et al., 2014, 2015; Griffing et al., 2015; DebRoy et al., 2016; Ison et al., 2016
Virulence gene profiles				X	Nandanwar et al., 2014; Manges et al., 2015
CRISPRs				X	Shariat and Dudley, 2014; Delannoy et al., 2015
Microarray	X	X	X	X	Liu and Fratamico, 2006; Hegde et al., 2013; Lacher et al., 2014
NeoSEEK^TM^ (PCR-mass spectroscopy)	X	X	X	X	Stromberg et al., 2015

*^a^The O-somatic and H-flagellar antigens define the *E. coli* serotype. Agglutination assays using antibodies that react with the specific O- and H-antigens are the basis for traditional serotyping. Molecular serotyping methods are generally based on genetic targets specific to the O- and H-antigens.*

Compared to phenotypic methods, genetic subtyping methods that are based on bacterial DNA, generally have better discriminatory ability. Of the various methods used for *E. coli* subtyping, PFGE is a reliable and highly discriminating method and has been considered to be the “gold standard” of typing methods. Through the establishment of PulseNet (Ribot et al., 2006), use of PFGE has had a major impact on pathogen subtyping and outbreak investigation.

In contrast to traditional serotyping, Luminex^®^-based suspension assays allow for simultaneous testing for multiple serogroups in a single assay. Lin et al. (2011) performed PCR assays targeting the *wzx* and *wzy* genes of ten Shiga toxin-producing *E. coli* (STEC) serogroups, and then used the Luminex^®^ system to identify the 10 serogroups through binding of the PCR products to fluorescent microspheres conjugated to specific DNA probes for each of the ten serogroups. Clotilde et al. (2015) used the Luminex^®^ technology, both antibody- and multiplex PCR-based, and compared them to traditional *E. coli* serotyping. The results of the two Luminex^®^ assays were mostly consistent, and 11 STEC isolates that were previously untypeable by traditional serotyping were able to be typed.

Multiplex PCR-based assays targeting unique regions within the *E. coli* O-AGCs have been used to determine the O-groups. A review by DebRoy et al. (2011) describes many of these assays, most of which target the *E. coli wzx* and *wzy* genes. Based on O-AGC sequence data for all O-groups, Iguchi et al. (2015b) designed 162 PCR primer pairs for identification and classification of *E. coli* O-serogroups. The primer pairs were used in 20 separate multiplex PCR assays with each assay containing 6–9 primer pairs that amplified products of different sizes so that they could be distinguished. A high-throughput PCR method based on the GeneDisc^®^ array targeted virulence genes and O- and H-type-specific genes for identification of STEC associated with severe illness (Bugarel et al., 2010b). Another high-throughput method, known as the BioMark^TM^ real-time PCR system (Fluidigm), used a panel of virulence genes as discriminative markers to differentiate EHEC O26 strains, EHEC-like O26 pathogenic strains, and avirulent O26 strains (Bugarel et al., 2011a).

Clustered regularly interspaced short palindromic repeats (CRISPR) are short, highly conserved DNA repeats separated by unique sequences of similar length, and they have been used for subtyping, identification, and detection of bacteria (Shariat and Dudley, 2014). Based on spacer content or sequencing of CRISPR loci, CRISPR-based typing analyses can be used to differentiate strains for epidemiological investigations or for detection. Delannoy et al. (2012) utilized CRISPR loci of seven important EHEC serotypes to develop real-time PCR assays, generating results based on CRISPR polymorphisms that correlated with specific EHEC O:H serotypes and the presence of EHEC virulence genes.

DNA microarrays have also been developed for molecular serotyping of *E. coli* (Liu and Fratamico, 2006; Ballmer et al., 2007; Geue et al., 2014; Lacher et al., 2014). One microarray method to identify *E. coli* serogroups involved spotting O-group-specific *wzx* or *wzy* gene oligonucleotides or PCR products onto the chip and hybridized with labeled PCR products of the entire O-AGCs (Liu and Fratamico, 2006). Lacher et al. (2014) reported on the use of an FDA-ECID (*E. coli* identification) microarray for O- and H-typing of *E. coli*. The ECID chip was designed based on >250 *E. coli* genomes and incorporates over 40,000 *E. coli* genes, including O- and H-group-specific genes, and approximately 9800 single nucleotide polymorphisms (SNPs). Antibody-based microarrays have also been developed to detect important non-O157 STEC serogroups (Gehring et al., 2013; Hegde et al., 2013). Although this method is rapid and has the potential to be used for high throughput screening, the utilization of this method is dependent on the availability of antibodies with good specificity.

The commercial introduction of next-generation sequencing technologies has made it possible to perform routine WGS of *E. coli* and other bacteria relatively rapidly and at affordable costs (Franz et al., 2014). Since WGS typing has discriminatory power superior to other typing methods, it has the potential to revolutionize bacterial subtyping. A MLST webserver was designed to determine sequence types (STs) of bacteria using WGS data. STs were determined from uploaded preassembled complete or partial genome sequences or short sequence reads obtained from different sequencing platforms (Larsen et al., 2012). Based on SNPs observed from WGS data, Norman et al. (2015) identified unique STEC O26 genotypes in human and cattle strains. These isolates had similar virulence gene profiles and did not cluster in separate polymorphism-derived genotypes, and thus human and cattle strains could not be distinguished within the phylogenetic clusters. An approach based on targeted amplicon sequencing for SNP genotyping was used to determine the relationship of *stx*-positive and *stx*-negative *E. coli* O26:H11 strains from cattle compared to the genomes of human clinical isolates (Ison et al., 2016). Joensen et al. (2015) described SerotypeFinder, a publicly available web tool hosted by the Center for Genomic Epidemiology, Denmark, which enables WGS-based serotyping of *E. coli.* Typing is based on *wzx, wzy, wzm*, and *wzt*, as well as flagellin-associated genes. Similar to SerotypeFinder, the VirulenceFinder tool can be used to determine virulence genes in *E. coli* to determine different pathogenic groups (Joensen et al., 2014).

Whole genome sequencing typing has the potential to be the new “gold-standard” for pathogen subtyping. However, some challenges need to be addressed before standardization and full implementation of this technology. The bioinformatic analyses required to analyze enormous amounts of sequence data generated by WGS are necessitating the development of analysis pipelines to enhance the assembly, annotation, and interpretation of the data, which will require a coordinated international approach (Franz et al., 2014; Oulas et al., 2015). Currently, the following databases for WGS and advanced detection are available: the 100K Genome Project^1^, GenomeTrakr Network^2^, Global Microbial Identifier^3^, and Advanced Molecular Detection^4^. These databases are creating a vast resource of microbial genome information for WGS-based surveillance of microbial pathogens. Furthermore, detailed analysis of WGS data can determine the *E. coli* O- and H-type and provide information on the resistome (antibiotic resistance gene profile) of the isolate, and the presence of specific virulence genes, prophages, and plasmids, as well as other genetic information important to identify *E. coli* pathotypes as well as utility in evolutionary studies. The advantages of WGS approaches are being recognized by academic, government, industry, and the private sector for addressing regulatory and public health needs. However, as we move toward the use of these genetic approaches for non-culture-based detection, characterization, subtyping, trace backs, and outbreak investigations, it will be critical to establish bioinformatics pipelines that are capable of analyzing and handling the large amounts of data that are generated.

## Author Contributions

PF, CD, YL, DN, GB, and PF have made a substantial, direct, and intellectual contribution to the work and approved it for publication.

## Conflict of Interest Statement

The authors declare that the research was conducted in the absence of any commercial or financial relationships that could be construed as a potential conflict of interest.

## Footnotes

1 http://100kgenome.vetmed.ucdavis.edu/

2 http://www.fda.gov/Food/FoodScienceResearch/WholeGenomeSequencingProgramWGS/

3 http://www.cdc.gov/amd/project-summaries/index.html

4 http://www.globalmicrobialidentifier.org/
